# Experimental Investigation and Artificial Intelligence-Based Modeling of Novel Biodiesel Fuels Containing Hybrid Nanoparticle Additives

**DOI:** 10.3390/molecules31060992

**Published:** 2026-03-16

**Authors:** Muhammed Mustafa Uyar, Ahmet Beyzade Demirpolat, Aydın Çıtlak

**Affiliations:** 1 Department of Motor Vehicles and Transportation Technologies, Vocational School of Arapgir, Malatya Turgut Özal University, Malatya 44800, Türkiye; muhammed.uyar@ozal.edu.tr; 2 Department of Electronics and Automation, Vocational School of Arapgir, Malatya Turgut Özal University, Malatya 44800, Türkiye; ahmetb.demirpolat@ozal.edu.tr; 3 Faculty of Engineering, Mechanical Engineering, Fırat University, Elazığ 23200, Türkiye

**Keywords:** hybrid NiO–SiO_2_ nanoparticles, diesel fuel, biodiesel, artificial intelligence modeling

## Abstract

This work investigates the influence of hybrid NiO–SiO_2_ nanoparticles on the engine behavior of biodiesel derived from waste sunflower oil and evaluates the experimental outcomes using a data-driven modeling approach. Biodiesel was produced via transesterification and doped with nanoparticles at concentrations of 50, 75, and 100 ppm. Performance and emission tests were conducted on a single-cylinder diesel engine operating at constant speed under varying loads. Specific fuel consumption, brake thermal efficiency, CO, HC, NO_x_, smoke opacity, and exhaust gas temperature were recorded and analyzed. The incorporation of nanoparticles improved combustion quality and contributed to substantial reductions in harmful emissions. The WSOB20 blend containing 100 ppm NiO–SiO_2_ provided the most balanced results, decreasing CO, HC, and smoke emissions by 39.50%, 39.40%, and 35.20%, respectively, relative to diesel fuel, while preserving competitive thermal efficiency. A linear regression model developed for CO prediction produced a low mean squared error (1.08 × 10^−5^), indicating strong predictive capability. The findings confirm that hybrid nanoparticle additives can enhance biodiesel performance while supporting accurate emission forecasting.

## 1. Introduction

Every year, the world seems to need a little more energy as industry grows, cities get bigger, and the population keeps increasing. Most of this demand is still covered by fossil fuels, which leads to problems such as air pollution, greenhouse gas emissions, and climate change. Because of these issues, there is growing interest in cleaner and more sustainable fuels that can replace at least part of conventional diesel. Biodiesel, which can be produced from vegetable oils, animal fats, or waste cooking oils, is one of the main alternatives. Biodiesels have several advantages over diesel fuels, including a longer lifespan, naturally fewer parts, lower emissions, and, most importantly, they are a superior energy source. In addition, biodiesel fuels have some potentially negative characteristics, such as high viscosity and density. In some operating conditions, this means the combustion does not work at its best, so specific fuel consumption can go up, and a small loss in performance can be seen. Also, the type of oil used as feedstock and the way the fuel is produced can cause extra issues with cold flow behavior and how stable the fuel is during storage. For these reasons, the use of nanoparticle additives to improve fuel properties and combustion behavior has become an important research topic. Nanoparticles can act as catalysts or oxygen carriers in the combustion process and help the fuel oxidize more completely. The appropriate distribution of metal oxide nanoparticle additives shortens the ignition delay in biodiesel-diesel blends, resulting in positive effects on flame temperature and the development of smaller fuel droplets. In many studies, these effects are associated with higher brake thermal efficiency (BTE) and lower CO, HC, and smoke emissions. However, the type, size, and concentration of nanoparticles must be selected carefully, because too high a dosage or unsuitable particles may cause agglomeration, injector clogging, or extra pumping work due to changes in fuel properties. Among different nanoparticle types, silicon dioxide (SiO_2_) and nickel oxide (NiO) are of special interest for biodiesel applications. SiO_2_ nanoparticles are chemically stable and relatively inexpensive. When added to the fuel, they can change physical properties such as viscosity and surface tension, which are important for atomization, and they can also provide extra reactive surface area in the combustion chamber. NiO nanoparticles are known as active oxidation catalysts and can promote the oxidation of carbonaceous species and incomplete combustion products. The accumulation of NiO nanoparticles, combustion time, and the pattern of heat release during combustion can be determined. Comparing SiO_2_ and NiO side by side as additives in biodiesel blends can therefore give useful information on which nanoparticle type is more suitable for cleaner and more stable combustion. Several experimental and review studies have investigated nanoadditives in biodiesel-fueled engines. Srinidhi et al. added 25–100 ppm NiO to a Neem biodiesel–diesel (NBE25) blend in a single cylinder VCR diesel engine and reported that advancing the injection timing and increasing the NiO concentration improved BTE and reduced specific fuel consumption, while NO_x_ emissions tended to rise with higher cylinder temperature [1]. Ansari et al. carried out a wide review of studies where CeO_2_, TiO_2_, SiO_2_, ZnO, NiO, CNT, and MWCNT were used as additives in biodiesel and biodiesel–diesel blends. From these works, they noted that nano additives in general tend to increase BTE, reduce BSFC (Brake Specific Fuel Consumption), and lower CO and HC emissions, while the change in NO_x_ depends mainly on the nanoparticle type and how much of it is added [2]. Campli et al. examined a Neem biodiesel–diesel (NB25) blend with 25–100 ppm NiO in a variable compression ratio CI engine and found that especially 75 ppm NiO increased BTE, decreased BSFC, and reduced NO_x_ emissions, with NiO acting both as a catalyst and as a heat sink [3]. Other studies focused more on SiO_2_ and mixed metal oxide additives. For example, Kumar et al. tested a chicken fat methyl ester (CFME)-based B20 blend containing 100 ppm SiO_2_ and 100 ppm NiO_2_ and reported that both nano additives reduced BSFC and that SiO_2_ provided more noticeable improvement in NO_x_, CO, and HC emissions [4]. Doğan et al. added green-synthesized SiO_2_ and TiO_2_ to safflower biodiesel blends and observed lower CO and HC emissions, a partial increase in NO_x_, and, in some cases, higher fuel consumption, but overall better environmental performance [5]. Several other works with SiO_2_ in different biodiesel systems (corn oil, sea mango methyl ester–isobutanol mixtures, and bio silica from waste oils) also reported improvements in efficiency and reductions in CO, HC, smoke, and sometimes NO_x_ [6,7,8,9,10]. In addition, Ghanati et al. reviewed recent studies on SiO_2_ and TiO_2_ additives and pointed out that these nanoparticles can improve viscosity, density, and cetane number, shorten ignition delay, and often reduce CO, HC, NO_x_, and particulate emissions, while environmental and health aspects still need attention [11]. The choice of biodiesel feedstock plays a key role in fuel quality, cost, and sustainability [12]. By sourcing used sunflower oil as a raw material, a resource that would otherwise be classified as waste, has been put to good use. Compared to edible oils used directly for food, waste oils also reduce food–fuel competition and offer a more sustainable approach [13]. Sunflower oil biodiesel is generally known to have acceptable fuel properties, and converting waste sunflower oil into biodiesel provides both economic and ecological benefits [14]. Over the past decade, many researchers have explored the use of nanoparticles to improve biodiesel combustion. However, an important question still remains: how can combustion performance be enhanced without creating new technical or environmental drawbacks? While nano additives are known to promote oxidation reactions and improve thermal efficiency, they may also influence fuel stability, cause particle clustering, or affect NO_x_ formation. These tradeoffs are not yet fully clarified. In addition, most previous studies have examined a single nanoparticle type and often relied on biodiesel produced from fresh vegetable oils. Direct comparisons between different metal oxide nanoparticles under the same engine conditions are relatively limited. This is especially true for biodiesel derived from waste sunflower oil, where systematic evaluations of both SiO_2_ and NiO—either separately or in combination—are still scarce. For this reason, the present study focuses on understanding how SiO_2_ and NiO nanoparticles influence combustion and emission behavior in waste sunflower oil biodiesel. By comparing their individual and hybrid effects under identical test conditions, this work aims to provide clearer insight into additive selection for sustainable and practical biodiesel applications. In this context, the main objective of the present study is to produce biodiesel from waste sunflower oil and to investigate the effects of SiO_2_ and NiO nanoparticle additives on the performance and emission characteristics of a diesel engine fueled with this biodiesel and its blends. For this purpose, waste sunflower oil biodiesel is first produced, and its fuel properties are determined. In the next stage, different amounts of SiO_2_ and NiO nanoparticles were added to prepare various fuel samples. These samples were subjected to engine tests under different operating conditions. In the study, the effects of SiO_2_ and NiO nanoparticle additions, separately and in hybrid blends, on the power of waste-based biodiesel fuels under the same test conditions are evaluated to ensure sustainable utilization of waste sunflower oil and cleaner combustion. In the literature, particularly for biodiesel produced from waste and vegetable oils, studies combining nanoparticle additives with AI/ML-based optimization methods have recently attracted attention [15,16,17]. In these studies, biodiesel derived from economical vegetable and waste oils was enhanced through hybrid nanoparticle addition, and suitable additive ratios to reduce emissions and improve combustion were identified using regression and learning based approaches. Thus, cleaner combustion, optimized fuel properties, and reduced emissions were targeted.

## 2. Results and Discussion

### 2.1. Evaluation of Fuel Consumption Performance

**Figure 1:**
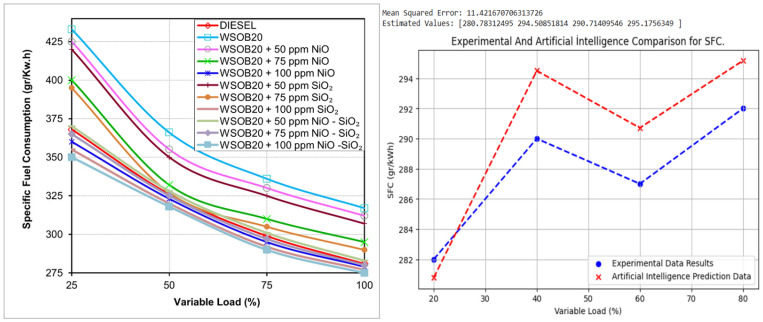
Load-dependent specific fuel consumption trends obtained from experimental measurements and artificial intelligence modeling.

Figure 1 illustrates how the specific fuel consumption of the tested fuels changes with operating conditions. The results suggest that the inclusion of SiO_2_ and NiO nanoparticles positively alters the fuel consumption behavior of biodiesel blends. Compared to the base fuel, nanoparticle-enhanced fuels exhibit lower SFC values, indicating a more efficient conversion of fuel energy into useful work. The WSOB20 + 100 ppm NiO–SiO_2_ blend delivered the most favorable results in terms of fuel consumption, whereas the highest SFC values were associated with the nanoparticle-free WSOB20 fuel. As engine load increased, SFC values generally declined, which can be explained by improved combustion stability and thermal performance at higher loads. Although biodiesel fuels are inherently prone to higher fuel consumption due to their physical properties, the addition of NiO–SiO_2_ nanoparticles was found to significantly reduce this limitation [18].

### 2.2. Thermal Efficiency Characteristics Under Varying Load Conditions

**Figure 2:**
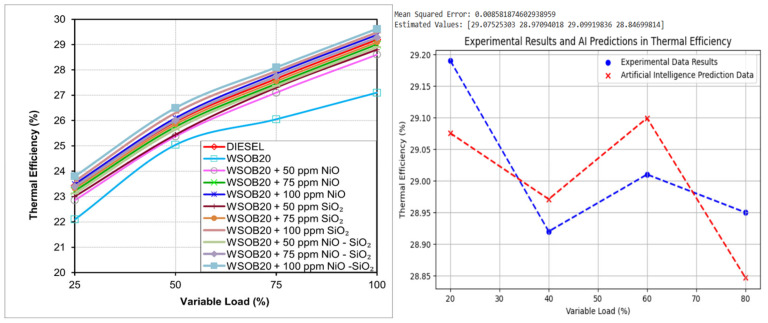
Experimental and artificial intelligence-based evaluation of thermal efficiency performance.

Thermal efficiency was assessed using the operational data recorded during the engine experiments. As shown in Figure 2, an overall increase in thermal efficiency was observed with rising load levels. To obtain consistent results, each test condition was repeated several times, and the average of the stable measurements was considered. The calculated efficiency values at four load conditions are presented in graphical form. A comparison among the tested fuels reveals that the WSOB20 blend produced the lowest thermal efficiency, whereas the WSOB20 + 100 ppm NiO–SiO_2_ blend achieved the highest performance in this respect. Moreover, several nanoparticle-enhanced fuels exceeded the thermal efficiency of conventional diesel, particularly WSOB20 + 100 ppm NiO, WSOB20 + 100 ppm SiO_2_, WSOB20 + 100 ppm NiO–SiO_2_, and WSOB20 + 75 ppm NiO–SiO_2_. These improvements can be linked to differences in both energy content and flow characteristics of the fuels. High viscosity, which is a common drawback of biodiesel fuels, is known to impair atomization and has a negative impact on thermal efficiency [19].

### 2.3. NO_x_ Emission Performance of Tested Fuels

**Figure 3:**
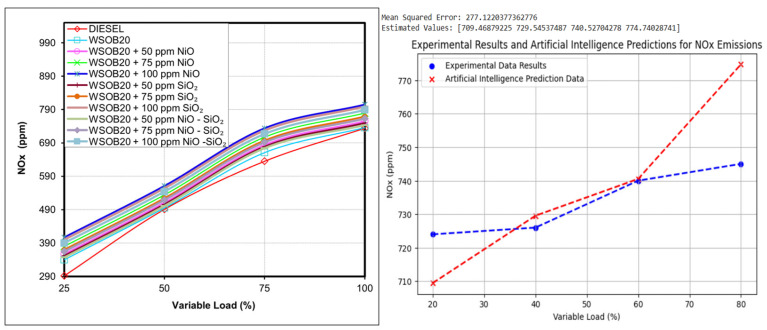
Experimental and artificial intelligence modeling of load-dependent variation in NOx values.

NO_x_ emissions are generally associated with severe combustion conditions, especially high temperatures and pressures inside the cylinder. In addition to these factors, changes in ignition delay and combustion duration may further intensify NO_x_ formation. The NO_x_ emission values obtained for the different test fuels are illustrated in Figure 3. The experimental results show that diesel fuel produced the lowest NO_x_ emission levels, whereas the highest NO_x_ values were observed for the WSOB20 + 100 ppm NiO blend. The main reason for this increase is that biodiesel fuels have a higher oxygen content. The increased amount of oxygen raises the combustion temperature and consequently increases NO_x_ formation [20].

### 2.4. Effect of Fuel Composition on CO_2_ Emissions

**Figure 4:**
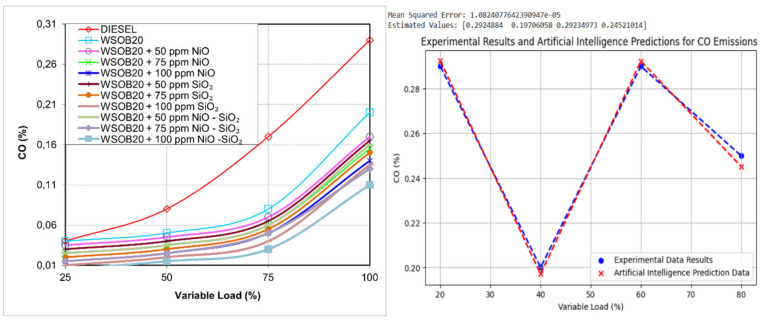
Experimental and artificial intelligence modeling of load-dependent variation in CO values.

Figure 4 presents the CO_2_ emission values measured during the experimental tests. According to existing literature, fuels characterized by low oxygen availability generally result in increased CO_2_ emissions due to less efficient combustion [21]. Consistent with this understanding, diesel fuel produced the highest CO_2_ emissions in the current study, while the lowest values were obtained from the WSOB20 + 100 ppm NiO–SiO_2_ blend. As the proportion of biodiesel in the fuel mixture increased, a noticeable reduction in CO_2_ emissions was observed. This behavior is mainly associated with the oxygen-rich nature of biodiesel fuels [21]. In addition, the use of nanoparticle additives further supported this reduction, indicating that nanoparticles contribute to improved combustion conditions within the engine cylinder.

### 2.5. Experimental Assessment of HC Emissions

**Figure 5:**
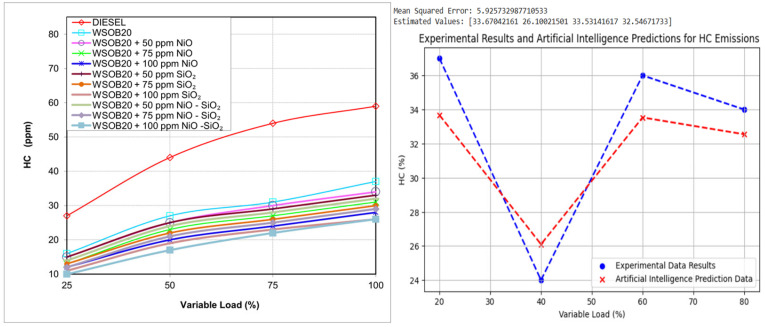
Experimental and AI-based modeling of load-dependent variations in HC emissions.

The HC emission characteristics of the tested fuel samples are shown in Figure 5. HC emissions are known to originate from incomplete combustion processes as well as from unburned gases remaining in the combustion chamber. According to the experimental findings, the highest HC emission levels were obtained with diesel fuel, whereas the lowest values were recorded for the WSOB20 + 100 ppm NiO–SiO_2_ fuel blend. The lower HC emissions associated with biodiesel-containing fuels can be explained by their higher cetane number and elevated oxygen content, which contribute to more complete combustion [21]. This trend is consistent with the observed increase in exhaust gas temperature as the biodiesel ratio increases. Furthermore, the addition of NiO–SiO_2_ nanoparticles provided an extra reduction effect on HC emissions.

### 2.6. Effect of Fuel Formulation on Smoke Emissions

**Figure 6:**
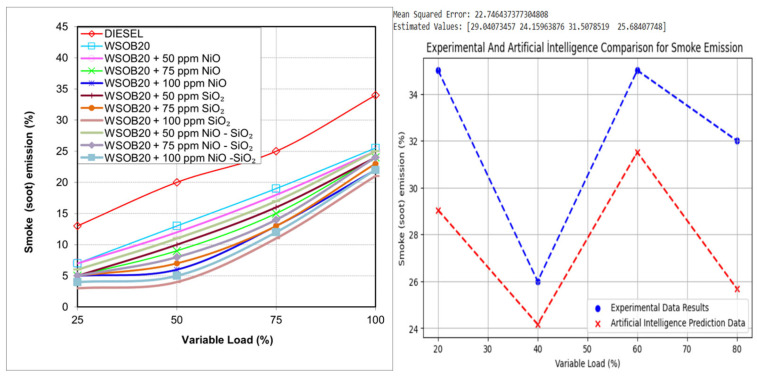
Comparison of smoke emission levels under different load conditions.

The smoke emission results obtained from the experimental tests are illustrated in Figure 6. Smoke formation is commonly associated with elevated flame temperatures and insufficient air–fuel mixing under turbulent combustion conditions [22]. According to the measured data, diesel fuel produced the highest smoke emission levels, whereas the lowest values were recorded for the WSOB20 + 100 ppm SiO_2_ fuel blend. Overall, a clear decreasing trend in smoke emissions was observed as the biodiesel content in the fuel increased. The incorporation of NiO–SiO_2_ nanoparticles contributed to a further reduction in smoke formation. However, when the additives were evaluated individually, SiO_2_ alone was found to be more effective than the hybrid nanoparticle combination in suppressing smoke emissions.

### 2.7. Effect of Fuel Composition on Exhaust Gas Temperature

**Figure 7:**
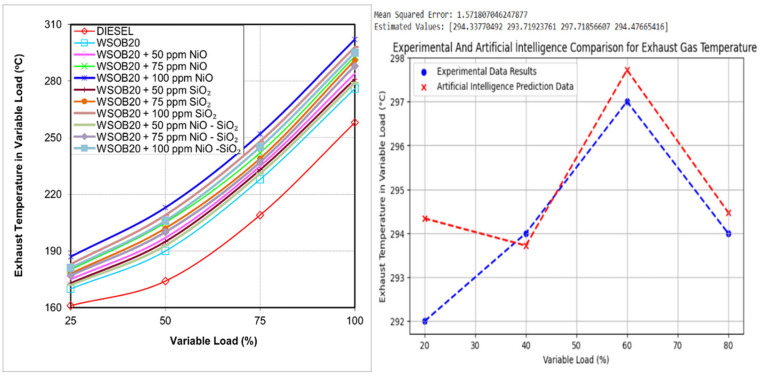
Comparison of exhaust gas temperature values for tested fuels.

Figure 7 presents the exhaust gas temperature data measured during the experimental tests. The findings indicate that diesel fuel produced the lowest exhaust temperatures, while the WSOB20 + 100 ppm NiO mixture resulted in the highest values. This rise in exhaust gas temperature can be associated with the higher oxygen availability and increased cetane number of biodiesel blends, which enhance combustion efficiency and raise cylinder temperatures [23].

## 3. Materials and Methods

This section summarizes the modular setups required to modify the fuels produced at this stage, the fuel sample preparation steps, and the systematic progress of the artificial intelligence-assisted evaluation process. Fuel samples were prepared by converting waste sunflower oil into biodiesel using the transesterification method, which is a well-established and widely applied technique for biodiesel production from vegetable and waste oils [24]. During production, Silicon Dioxide (SiO_2_) and Nickel Oxide (NiO) nanoparticles were evaluated heterogeneously as additives to support the process and facilitate product separation, in line with approaches reported in previous nanoparticle-assisted biodiesel studies [25]. The biodiesel obtained after the reaction was retained in a vacuum evaporator to remove moisture and residual methanol. The gradually applied low pressure and mild heating enabled effective evaporation of moisture while helping to protect free fatty acids (FFAs) from degradation, as commonly recommended in biodiesel post treatment procedures [25]. In the experimental procedure, waste sunflower oil was selected as the base material. Methanol (≥99.8%, Merck, Darmstadt, Germany) was utilized to drive the transesterification process, whereas sodium hydroxide (≥98%, Sigma-Aldrich, St. Louis, MO, USA) functioned as the catalyst. The SiO_2_ and NiO nanoparticles, obtained from Nanografi Nano Technology (Ankara, Turkey), possessed high purity levels of 99.5% and 99.9%, respectively, with particle sizes in the range of 10–35 nm. The morphological suitability of the nanoparticles was verified by SEM analysis. The produced fuel samples were analyzed under laboratory conditions and confirmed to comply with relevant ASTM biodiesel fuel standards [26]. To achieve adequate dispersion in biodiesel–nanoparticle mixtures, a two-stage mixing procedure was applied. Initially, an ultrasonic bath was used to reduce particle agglomeration, followed by a high-frequency homogenizer to enhance mixture homogeneity, which is a commonly adopted method for preparing stable nanoparticle blended fuels [27]. After producing hybrid SiO_2_–NiO modified biodiesel samples with different additive combinations, engine tests were conducted. A schematic representation and detailed description of the experimental setup are provided in Section 2.6. Engine performance and exhaust emission measurements were carried out using a single-cylinder, four-stroke, water-cooled diesel generator (Kama Reis KDK 7500 CE, Istanbul, Turkey) operated without any mechanical modifications. Prior to testing, the engine was run until steady state operating conditions were achieved, after which the prepared fuels were evaluated at load levels of 2, 4, 6, and 8 kW. To ensure measurement reliability, each experiment was repeated seven times, and consistent data were recorded. During transitions between different fuel samples, the fuel line was thoroughly cleaned to avoid cross-contamination. The experimental data were used to calculate brake thermal efficiency and specific fuel consumption (g/kWh), as well as to evaluate exhaust emissions, including CO, NO_x_, HC, exhaust gas temperature, and smoke opacity. Emission measurements were conducted using a BOSCH BEA 250 D exhaust gas analyzer (Stuttgart, Germany), while smoke opacity measurements were additionally verified using an AVL 437 smoke meter (Graz, Austria), as commonly applied in diesel engine emission studies [28]. Artificial intelligence tools were employed to analyze the experimental results more systematically. A linear regression model was developed using the dataset obtained from the experiments, as this method has been shown to provide reliable trend estimation for engine performance and emission parameters with limited datasets [29]. Experimental data processing, statistical evaluation, and model development were performed using Python in the Google Colab environment (Python 3.10).The outcomes of the regression analysis were evaluated together with trends reported in the literature to minimize discrepancies between experimental observations and predictive modeling. Considering the limited number of studies that combine experimental biodiesel–nanoparticle data with data-driven evaluation approaches, this study aimed to represent the experimental trends at an appropriate level using linear regression analysis.

### 3.1. Reaction Parameters

Biodiesel synthesis from waste sunflower oil was achieved via an alkaline transesterification process, which is a widely established method for biodiesel production from vegetable and waste oils [30]. Methanol was introduced as the alcohol source, whereas sodium hydroxide acted as the catalyst. The reaction was maintained at 60 ± 2 °C for 90 min with mechanical agitation at 600 rpm. A methanol to oil molar ratio of 6:1 was applied, and the catalyst loading was fixed at 1 wt% based on the oil mass. Following the reaction, the mixture was transferred to a settling stage and kept for 8 h to ensure complete phase separation. The upper biodiesel layer was separated, rinsed with distilled water to remove residual reactants and catalyst remnants, and subsequently dried under vacuum conditions to eliminate moisture. These operational parameters were defined after preliminary optimization studies aimed at maximizing conversion efficiency and preserving the stability of the produced fuel.

### 3.2. Fuel Sample Production

**Figure 8:**
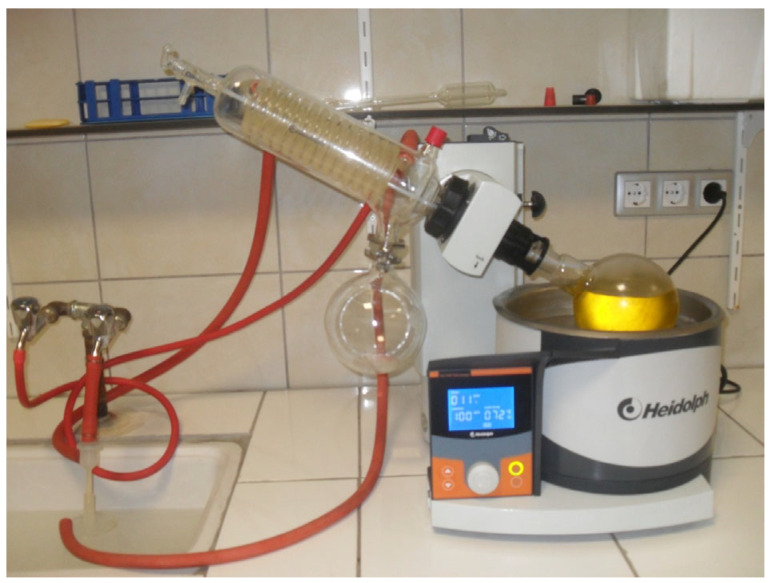
Evaporation process.

The production of biodiesel containing hybrid SiO_2_–NiO additives was carried out using transesterification, a commonly preferred method for processing waste sunflower oil [13]. During synthesis, the oil was reacted with methanol while sodium hydroxide was used as the catalyst under controlled operating conditions. After completion of the reaction, the mixture was transferred to a separatory funnel and allowed to rest until clear phase separation occurred and glycerin could be removed. The biodiesel phase was subsequently washed repeatedly with distilled water to remove impurities and catalyst residues. Finally, remaining moisture and free fatty acids were removed using an evaporator. A schematic view of the evaporator system applied in this final purification stage is provided in Figure 8.

**Figure 9:**
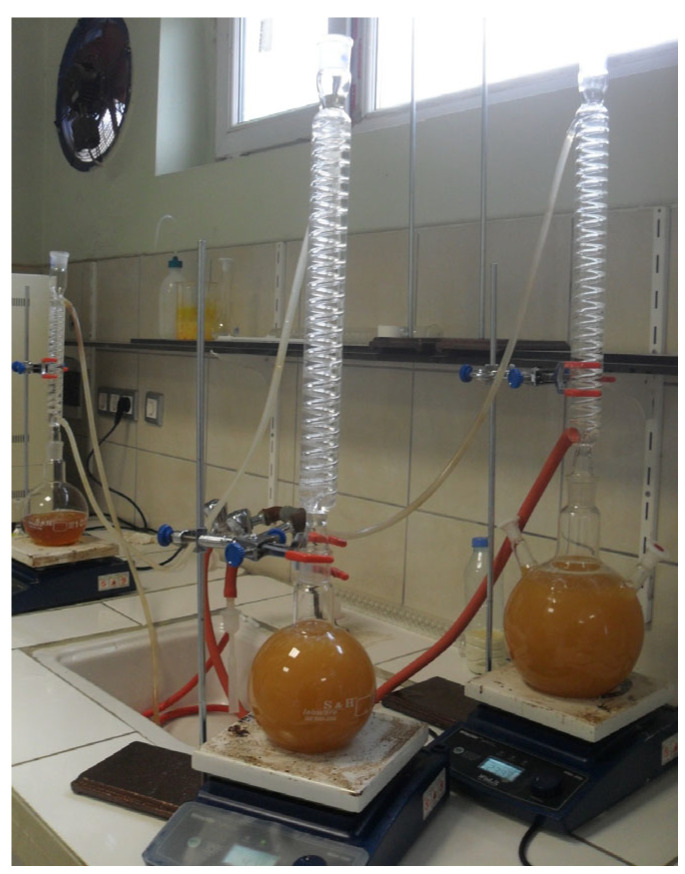
Transesterification reaction.

Waste sunflower oil was selected as the feedstock for biodiesel production. Prior to the transesterification process, the oil was heated to 60 °C. Separately, a reaction mixture consisting of methanol and sodium hydroxide was prepared. The methanol volume corresponded to 20% of the oil volume, and sodium hydroxide was added at a concentration of 0.4 wt% relative to the oil as the catalyst. After preparation, the oil and reaction mixture were combined and stirred continuously for 2 h to complete the transesterification reaction. A schematic overview of the biodiesel production process is presented in Figure 9.

**Figure 10:**
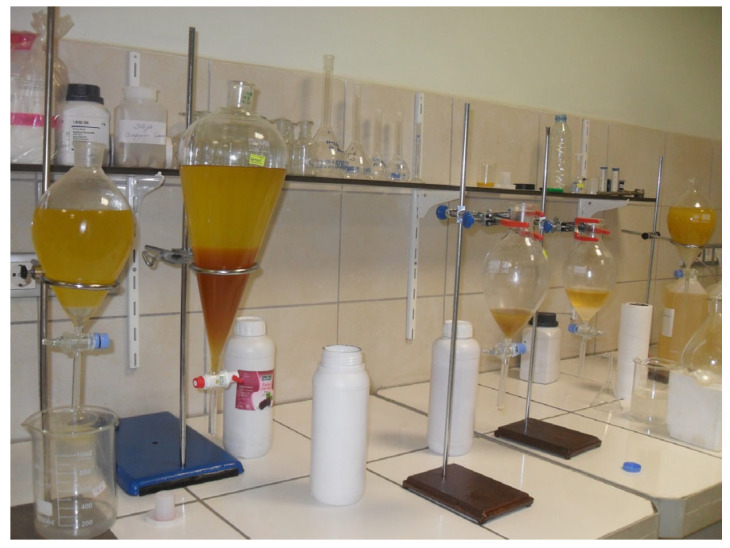
Phase separation and washing process.

Following completion of the transesterification reaction, the mixture was transferred to a separatory funnel and allowed to stand until clear phase separation occurred. The lower glycerin layer was removed, and the remaining biodiesel phase was washed with distilled water to obtain the final fuel product, as illustrated in Figure 10. In the subsequent step, vacuum evaporation was employed to eliminate residual moisture and complete the biodiesel purification process. For engine testing, the vegetable-derived biodiesel was blended with conventional diesel fuel at a volumetric ratio of 20:80, and the chemical properties of the prepared blends were determined under laboratory conditions. The waste sunflower oil biodiesel blend was identified as WSOB20. A schematic overview of these preparation steps is provided in Figure 10.

**Figure 11:**
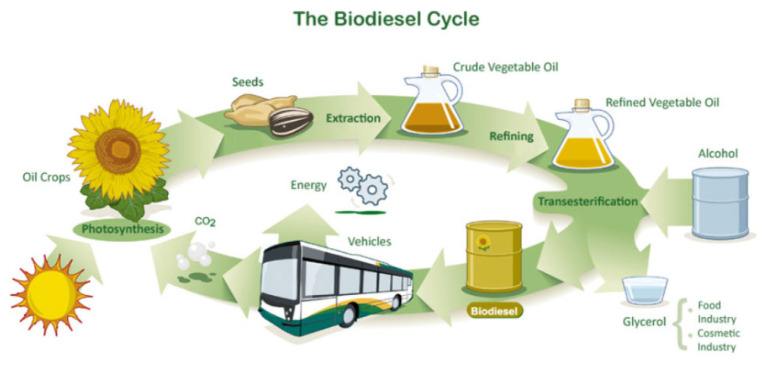
Schematic representation of the biodiesel production process [31].

In the final stage, an evaporation step was applied to complete biodiesel production and obtain the final fuel product. The biodiesel derived from waste sunflower oil was subsequently blended with conventional diesel fuel at a volumetric ratio of 20% (*v*/*v*). The chemical properties of the prepared fuel blend were determined under laboratory conditions. A schematic summary of these steps is presented in Figure 11.

### 3.3. Chemical Analysis of Fuel Samples

Laboratory-based analyses were performed to determine the chemical characteristics of the biodiesel fuel samples. All measurements were repeated to confirm consistency, and the observed variation between results remained within a 2% margin. The average values derived from these analyses are presented in Table 1.

### 3.4. Nanoparticle Addition to Fuel Samples

In this phase, the basic technical properties of the silicon dioxide (SiO_2_) and nickel oxide (NiO) nanoparticles considered in the study were evaluated. These nanoparticles were employed as heterogeneous catalysts by being directly added to the transesterification reaction during biodiesel production from waste sunflower oil. Their inclusion in the reaction medium was intended to promote higher triglyceride to methyl ester conversion, support easier phase separation, and enhance overall process efficiency while maintaining economic and environmental advantages. Furthermore, due to their high oxygen storage capacity, improved catalytic surface properties, and homogeneous dispersion capabilities at the nanoscale, SiO_2_ and NiO nanoparticles were also used as additives to biodiesel after production, as they improve combustion performance and contribute to the reduction in exhaust emissions [32]. The characterization studies conducted included the evaluation of technical parameters and the analysis of SEM images. NiO–SiO_2_ nanoparticles were chosen because they bring together complementary benefits for biodiesel performance. NiO serves as an oxidation catalyst, enhancing combustion and reducing incomplete combustion products, while SiO_2_ is stable, inexpensive, and helps improve fuel atomization by adjusting viscosity and surface tension. Combining these two nanoparticles allows us to take advantage of both chemical and physical enhancements, creating a synergistic effect that improves thermal efficiency and lowers emissions. While other nanoparticle combinations, such as NiO–TiO_2_ or ZnO–SiO_2_, exist in the literature, NiO–SiO_2_ was considered a practical and cost-effective choice for waste sunflower oil biodiesel. The results obtained revealed that the SiO_2_ and NiO nanoparticles used possess suitable qualities for the application. Furthermore, the production process of SiO_2_ and NiO modified biodiesel fuels is described in detail, step by step, in this study. NiO–SiO_2_ nanoparticles were obtained from a commercial supplier and used as received. The incorporation of these nanoparticles into waste sunflower oil biodiesel was performed in our laboratory to produce the nanoparticle-enhanced biodiesel blends. The study focused primarily on evaluating the effects of these additives on emission reduction and thermal performance rather than on cost or large-scale production.

#### 3.4.1. SiO_2_ and NiO Particle Technical Specifications

The technical properties of SiO_2_ and NiO nanoparticles are shown in Table 2.

#### 3.4.2. SiO_2_ and NiO Nanoparticle SEM Image

**Figure 12:**
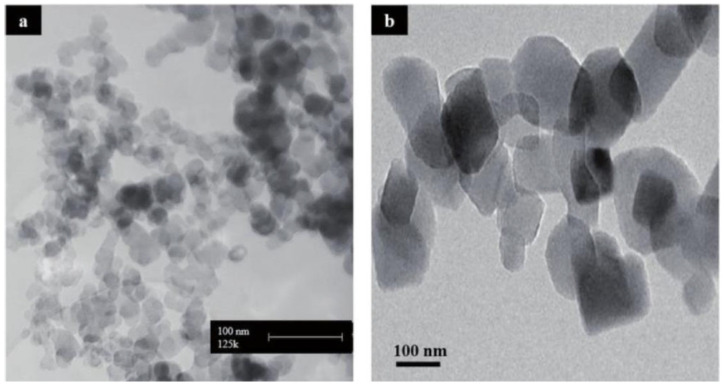
SEM images of Silicon Dioxide (SiO_2_) nanoparticles: (**a**) SEM image showing the overall distribution and agglomerated structure of the SiO_2_ nanoparticles; (**b**) higher-magnification SEM image illustrating the particle morphology and approximate nanoscale dimensions. Scale bar: 100 nm.

**Figure 13:**
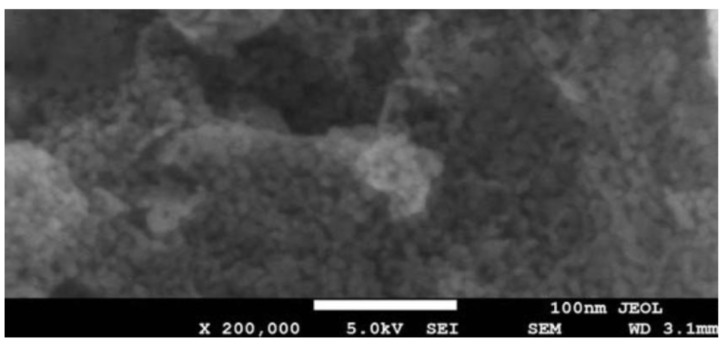
SEM image of Nickel Oxide (NiO) nanoparticles.

For the experimental work, silicon dioxide (SiO_2_) and nickel oxide (NiO) nanoparticles were sourced from Nanografi Nano Technology located in Ankara, Türkiye. The assessment of nanoparticle surface morphology and structural features was carried out using manufacturer-provided scanning electron microscopy (SEM) images. Based on the SEM observations presented in Figure 12 and Figure 13, both SiO_2_ and NiO nanoparticles display an almost spherical shape, a uniform spatial distribution, and a very low tendency toward particle agglomeration. These morphological features are consistent with previously reported characteristics of SiO_2_ and NiO nanoparticles in similar fuel-related applications [33]. The measured particle sizes meet the nanoparticle criteria. They are similar to the properties reported by the supplier and to previously published data in the literature for SiO_2_ and NiO nanoparticles. Morphological findings reveal that the nanoparticles maintain a high specific surface area and structural integrity. These properties offer significant advantages in terms of enhancing surface interactions when added as additives to biodiesel fuels. Consequently, the data confirm that the structural properties of SiO_2_ and NiO nanoparticles are consistent with those reported in similar studies in the literature.

The analyses performed revealed that the nanoparticles are in the approximately 100 nm size range and exhibit a high degree of crystalline properties. This size distribution was found to be consistent with the technical analysis results provided by the manufacturer and the morphological data obtained from SEM images. The data obtained show that the applied characterization methods yielded consistent results. Both SiO_2_ and NiO nanoparticles were found to possess clearly defined cubic crystal formations. The presence of a well-aligned nanoparticle structure is considered beneficial for catalytic processes, as it can intensify surface interactions and thereby contribute to more efficient combustion and lower emission formation when biodiesel fuels are utilized.

### 3.5. Biodiesel Blend with SiO_2_ and NiO Nanoparticles

**Figure 14:**
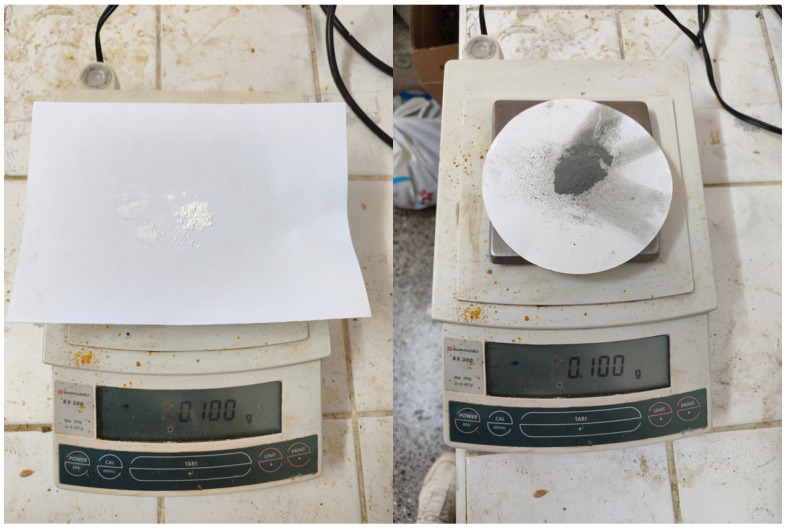
Digital weight measurement instrument. SiO_2_ nanoparticles are white, NiO nanoparticles are dark gray.

In this stage of the study, the produced bio-based and environmentally sustainable biodiesel was mixed with commercial diesel fuel at a 20% volumetric proportion. Silicon dioxide (SiO_2_) and nickel oxide (NiO) nanoparticles were separately added to the B20 fuel blends at concentrations of 50, 75, and 100 ppm. Furthermore, hybrid blends containing simultaneous additions of SiO_2_ and NiO at the same concentration levels were prepared and subjected to laboratory evaluation. All formulated fuel samples were found to meet ASTM fuel quality requirements [33], and the experimental program was continued accordingly. To ensure effective and stable dispersion of nanoparticles, the fuel blends were first treated in an ultrasonic bath and then processed using a high-frequency homogenizer. The required nanoparticle quantities at the ppm scale were determined with a precision balance, an image of which is provided in Figure 14.

**Figure 15:**
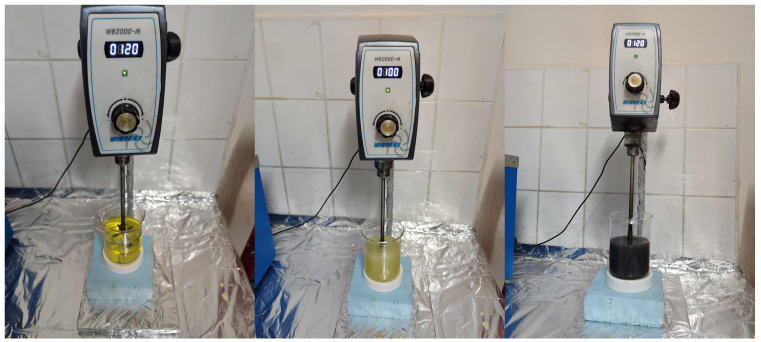
High-frequency homogenizer (HFC).

A high-frequency homogenizer was used to ensure the homogeneous distribution of SiO_2_ and NiO nanoparticles in the fuel. A homogenizer used to add SiO_2_ and NiO nanoparticle additives to fuel samples containing 20% biodiesel is shown in Figure 15.

**Figure 16:**
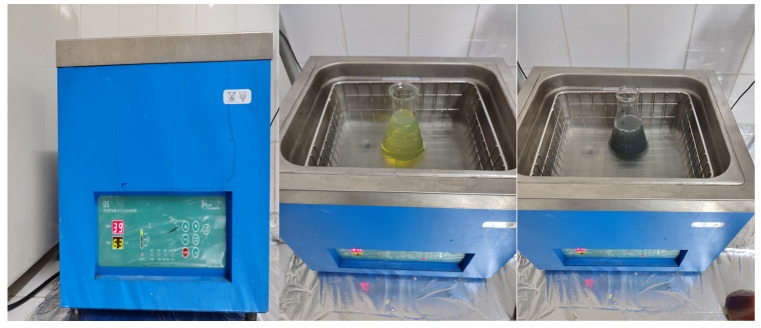
An experimental ultrasonic bath setup was utilized to promote stable and homogeneous distribution of nanoparticles in the fuel matrix.

To obtain a homogeneous mixture, the samples were first mixed using a homogenizer and then subjected to ultrasonic bath treatment. The ultrasonic bath operates on the principle of ensuring a more even distribution of nanoparticles within the mixture through high-frequency waves. In this process, the energy transferred to the fuel medium causes cavitation formation, and the vibrations resulting from the collapse of the microbubbles contribute to the effective mixing of the fuel with SiO_2_ and NiO nanoparticles [34]. The ultrasonic bath setup where fuel and hybrid SiO_2_–NiO nanoparticles are processed together is shown in Figure 16.

**Figure 17:**
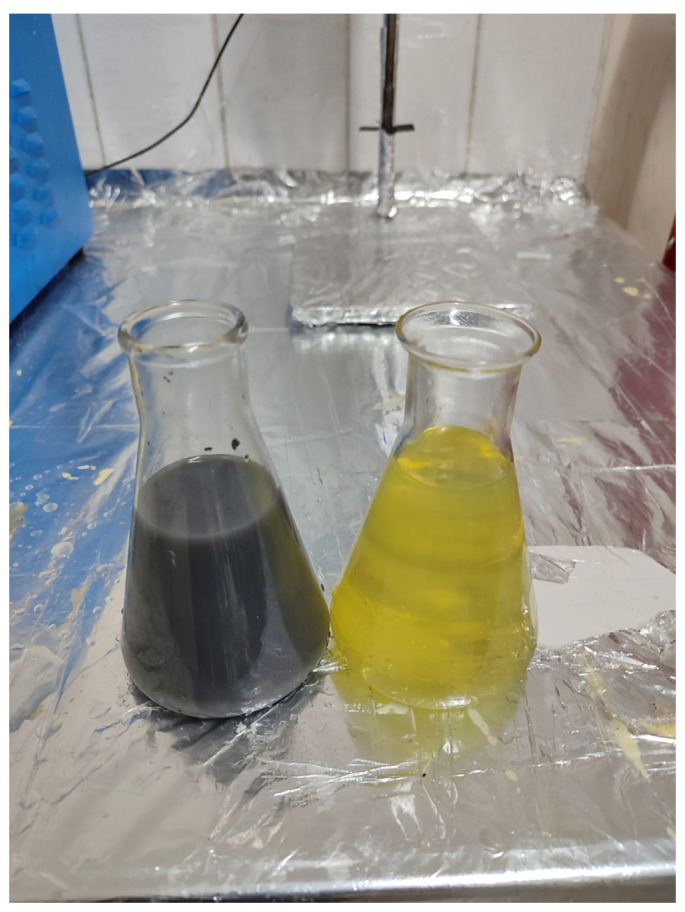
Visual appearance of the fuel samples containing nanoparticle additives at different compositions.

Figure 17 shows images related to the mixing of SiO_2_ and NiO nanoparticles with biodiesel fuel.

SiO_2_ nanoparticles are white, while NiO nanoparticles are dark gray. In biodiesel fuel, the white nanoparticles did not significantly alter the fuel’s color. However, when biodiesel fuel came into contact with the dark gray nanoparticles, it turned the fuel a darker color. Biodiesel obtained from waste sunflower oil was blended with diesel fuel at a ratio of 20% by volume. This mixture was defined as WSOB20 in this study. WSOB20 fuel was homogeneously mixed with SiO_2_ and NiO nanoparticles at concentrations of 50, 75, and 100 ppm using an ultrasonic bath method. In addition, hybrid fuel mixtures using SiO_2_ and NiO nanoparticles in the same ratios were also prepared [34]. The chemical behavior of the fuel samples produced in this study was evaluated through a series of laboratory-based analyses. The fuel mixtures used in the experiments were named as follows: WSOB20 + 50, 75, and 100 ppm SiO_2_, WSOB20 + 50, 75, and 100 ppm NiO, and WSOB20 + 50, 75, and 100 ppm SiO_2_–NiO.

### 3.6. Experiment Setup

**Figure 18:**
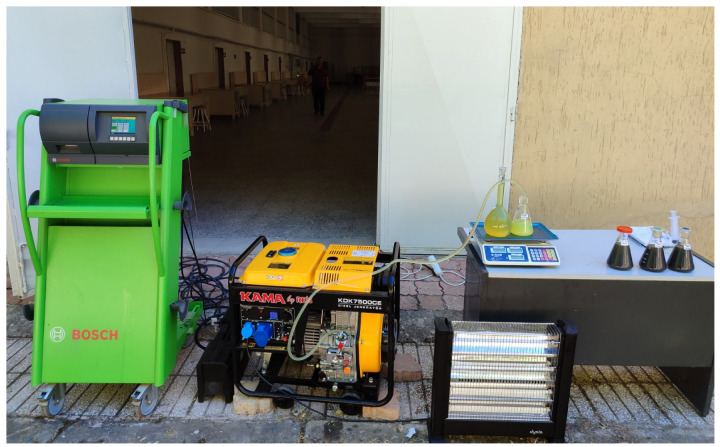
The experimental setup.

The experimental arrangement employed in this study is presented in Figure 18. All engine tests were conducted without introducing any structural or mechanical modifications to the test unit. Following the preparation of hybrid biodiesel fuels containing SiO_2_ and NiO nanoparticles derived from vegetable-based and waste sunflower oils, the fuels were evaluated under engine operating conditions. Before each test sequence, the engine was operated until steady-state conditions were achieved. The experiments were then carried out at load levels of 2.5, 5, 7.5, and 10 kW. For each fuel type, the tests were repeated three times, and the average values of the stabilized measurements were used for analysis. During the experiments, brake thermal efficiency, specific fuel consumption, CO, NO_x_, and HC emissions, exhaust gas temperature, and smoke opacity were recorded. To prevent cross-contamination between successive tests, the fuel system was thoroughly flushed before switching to a new fuel sample. All experimental measurements were performed using a domestically manufactured Kama Reis KDK 7500 CE diesel generator, produced by Kama by Reis Machinery Industry Co., Ltd. (Istanbul, Turkey), under standard operating conditions.

#### 3.6.1. Method Used for Calculations

##### Evaluation of Specific Fuel Consumption

The specific fuel consumption measured during the test process describes the relationship between the volume of fuel used and the power provided by the engine. After the experiments, the necessary calculations were carried out based on the method presented in Equations (1) and (2):

(1) $$B=\frac{500\times \rho_{y}\times 3.6}{∆t}$$

*B*: Denotes the mass-based fuel consumption rate expressed on an hourly basis (kg/h);
∆*t*: Represents the time interval required for the consumption of 500 mL of fuel;
*ρ*_y_: Refers to the density of the tested fuel sample (kg/L);

(2) $$b_{e}=\frac{B}{P_{e}}$$

*b_e_*: Is defined as the specific fuel consumption per unit output power (kg/kWh);
*P_e_*: Indicates the brake power produced by the engine (kW).

##### Thermal Efficiency

Thermal efficiency is defined as the proportion of useful power produced by the engine relative to the total energy input delivered through the fuel. In this study, the calculation of thermal efficiency was carried out using the formulation presented in Equation (3):

(3) $$\eta_{t}=\frac{3600N_{e}}{{BH}_{u}}=\frac{3600N_{e}}{{N_{e}b_{e}H}_{u}}=\frac{3600}{{b_{e}H}_{u}}$$

*N_e_*: Corresponds to the effective power delivered by the engine (kW);
*η_t_*: Expresses the overall thermal efficiency of the engine system;
*H_u_*: Corresponds to the lower calorific value characterizing the energy content of the fuel (kJ/kg).

#### 3.6.2. Error Analysis

Multiple experimental measurements were performed throughout the evaluation of the fuel samples. To evaluate the accuracy and consistency of these measurements, an uncertainty analysis approach was adopted. Factors including experimental conditions and the calibration quality of the measuring instruments were identified as the main contributors to measurement deviations. The overall experimental uncertainty (U, %) was determined using the root sum square method, in which the individual uncertainties of key performance parameters (e.g., $u_{SFC},u_{TE}$) were combined. The general expression employed for this analysis is provided in Equation (4):

(4) $$U=\sqrt{u_{SFC}^{2}+u_{TE}^{2}+u_{{NO}_{x}}^{2}+u_{CO}^{2}+u_{HC}^{2}+u_{SE}^{2}+u_{EGT}^{2}}$$

$$U=\sqrt{{\left(1.07\right)}^{2}+{\left(0.292\right)}^{2}+{\left(1\right)}^{2}+{\left(0.01\right)}^{2}+{\left(1\right)}^{2}+{\left(0.1\right)}^{2}+{\left(0.1\right)}^{2}}=\pm 1.803\%$$

### 3.7. Machine Learning System (MLS)

**Figure 19:**
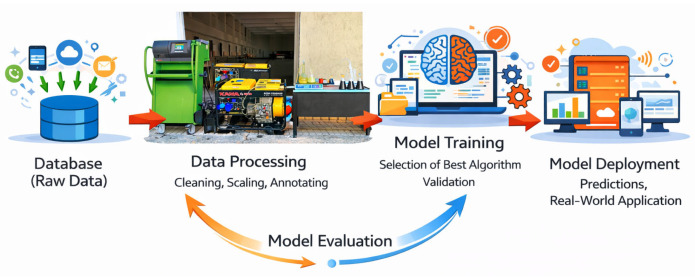
A schematic of the machine learning system process stages.

In biodiesel fuel production, data analysis-based methods have become widely used to achieve optimal results. Among these methods, machine learning-based modeling approaches stand out. Machine learning aims to develop predictive models from existing data by combining mathematical and statistical infrastructure through computer software. The modeling process relies on the combined evaluation of the dataset and the algorithm used. In this study, the linear regression method, one of the machine learning techniques, was used. Linear regression establishes the relationship between independent variables and the predicted value, enabling the calculation of the error rate [34]. A review of the existing literature reveals that studies on the machine learning supported analysis of the environmental and economic impacts of biodiesel fuel data are limited. Therefore, in our study, linear regression analysis was preferred to determine the extent to which the data of the fuel samples approached the optimum values. The general structure of the model used is shown in Figure 19.

**Figure 20:**
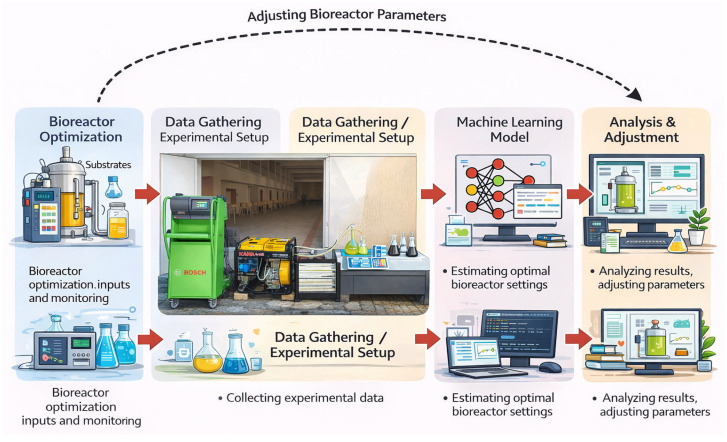
A graphic summary showing the stages of the study.

A graphical summary showing the stages of the study is shown in Figure 20.

### 3.8. Optimization and Artificial Intelligence Modeling

In order to interpret and predict the combined performance and emission response of biodiesel fuels modified with hybrid SiO_2_–NiO nanoparticles, a machine learning–assisted optimization strategy was adopted. Linear regression was chosen as the prediction model, as it provides an effective framework for analyzing continuous experimental datasets. The model was constructed using experimental parameters such as engine load, specific fuel consumption, exhaust gas temperature, CO, HC, and NO_x_ emissions, smoke opacity, and thermal efficiency. To avoid bias arising from differences in parameter scales, the dataset was normalized prior to modeling. The available data were divided into two groups, with 80% used for training the model and 20% employed for validation. The main objective of the modeling approach is to determine the optimum hybrid nanoparticle concentration that maximizes thermal efficiency while minimizing emissions and SFC. Linear regression results showed a high agreement between measured and predicted CO_2_ emissions (R^2^ = 0.956; MSE = 1.08 × 10^−5^). Furthermore, the model was found to successfully reflect the nonlinear emission trends that emerged with increasing nanoparticle concentration up to 100 ppm. Optimization results revealed that the WSOB20 + 100 ppm NiO–SiO_2_ fuel mixture is the most suitable option in terms of overall performance and emissions. The agreement of the modeling outputs with the experimental results demonstrates that the linear regression approach is a reliable tool for predicting biodiesel fuel behavior.

## 4. Conclusions 

The experimental evidence presented in this study shows that enriching waste sunflower oil biodiesel with hybrid NiO–SiO_2_ nanoparticles leads to measurable improvements in engine efficiency and exhaust quality. The uniform dispersion of mostly spherical particles below 100 nm, confirmed by SEM analysis, appears to support a more controlled and complete combustion process. This structural characteristic is directly reflected in engine performance outcomes. Among the tested fuels, the WSOB20 blend containing 100 ppm nanoparticles delivered the most consistent results. Under identical operating conditions, thermal efficiency increased by 3.50% and specific fuel consumption decreased by 2.20%, indicating a clearer conversion of fuel energy into useful work. Emission behavior showed even greater improvement. CO, HC, and smoke emissions declined by 39.50%, 39.40%, and 35.20%, respectively, demonstrating that incomplete combustion products were substantially reduced. Although a slight rise in NO_x_ emissions was observed, this increase does not offset the overall reduction in harmful exhaust components. The regression-based model used for CO_2_ prediction produced a very low mean squared error (1.08 × 10^−5^), confirming that computational tools can reliably support experimental findings. While longer-term durability and NO_x_ mitigation strategies should be examined in future research, the present data clearly indicate that combining nanotechnology with waste-derived biodiesel offers a practical route toward cleaner and more efficient diesel engine operation.

## Figures and Tables

**Table 1:** Summary of the chemical properties of the fuel samples produced.

Fuel Type	Density (g/cm^3^)	Viscosity (mm^2^/s)	Cetane Number (CN)	Lower Heating Value (kJ/kg)
Diesel Fuel	0.8370	3.321	51.50	42,816
%100 WSOB	0.8790	4.380	53.90	39,420
WSOB20	0.8455	3.534	52.00	42,139
WSOB20 + 50 ppm NiO	0.8594	4.132	53.55	42,528
WSOB20 + 75 ppm NiO	0.8597	4.149	53.65	42,788
WSOB20 + 100 ppm NiO	0.8603	4.178	53.80	43,084
WSOB20 + 50 ppm SiO_2_	0.8418	3.772	54.20	42,817
WSOB20 + 75 ppm SiO_2_	0.8432	3.805	54.35	42,987
WSOB20 + 100 ppm SiO_2_	0.8448	3.870	54.60	43,258
WSOB20 + 50 ppm NiO–SiO_2_	0.8508	4.173	54.80	42,830
WSOB20 + 75 ppm NiO–SiO_2_	0.8517	4.184	55.05	42,988
WSOB20 + 100 ppm NiO–SiO_2_	0.8528	4.205	55.65	43,307

**Table 2:** Technical properties of SiO_2_ and NiO nanoparticles.

SiO_2_ Information							
Name		SiO_2_					
CAS Number		7631-86-9					
Number		NG04SO3104					
Size		15–35 nm					
Purity Percentage		99.5+%					
**Test Results**							
		**Elemental Analysis (%)**					
Color	Specific Surface Area (m^2^/g)	Bulk Density (g/cm^3^)	True Density (g/cm^3^)	Fe	Ca	Ti	Na
White	150–550	<0.1	2.2	0.002	0.007	0.012	0.003
**NiO Information**							
Name		NiO					
CAS Number		1313-99-1					
Number		NG04SO2803					
Size		10–20 nm					
Purity Percentage		99.9%					
**Technical Properties**							
Color	Specific Surface Area (m^2^/g)	Loss on Ignition (LOI) @ 850 °C, 2 h (%)		True Density (g/cm^3^)		Appearance	
Dark Gray	80–100	1.5 max		2.2		Powder	

## Data Availability

The data that has been used is confidential.
